# Report on New Remedies

**Published:** 1868-04

**Authors:** J. W. H. Baker


### REPORT ON NEW REMEDIES.

BY J. W. H. BAKER, M. D.,

*Mr. Chairman, and Gentlemen of the Iowa State Med. Society:*

Having been appointed chairman of a committee to report upon the subject of *New Remedies*, I had thought to have made a verbal apology in the place of anything more tangible upon the matter. But after thinking of the duties of our members, and the result, were all our members of committees to offer such apologies, I within the past few days concluded to write a short report, hoping it will serve as my excuse from totally neglecting to write upon a matter of so little interest, it seems, that other members of this committee have neglected to aid me, by even suggestions, to fill a subject which, although extensive and useful, is difficult and at times repulsive. You will hardly expect from me a report upon one of the most difficult subjects in medical science which shall be in all its parts new, and free from the old; for I acknowledge myself sometimes slow to adopt the use of a new remedy. I well remember an eminent Surgical Professor in one of our Eastern Medical Colleges who adopted the use of ether as an anæsthetic agent soon after its discovery, and continued its use long after chloroform was used as its substitute. His reason, when asked why he did not use chloroform, was given in the short and pert adage of "let well enough alone;" but some time afterward, while performing an amputation, one of the physicians present observing that this same professor was using chloroform, he says to him: "Why, Doctor, I thought you never used chloroform?" The Professor's reply came up at once, and was this, viz: "I think I learn something new every year." So it is with many of us. While slow to adopt all the new remedies which present themselves, we think we learn something new every year; and perhaps some of us might acquire a new idea once in six months.

New remedies is the subject on which I am expected to report—new remedies for old and for new diseases—or I would rather express it by saying the new use of old remedies as applied to the treatment of disease. Formerly this insatiate appetite for new remedies existed among a class of men in search for the philosopher's stone, or of chemists engaged with their retorts in seeking a method of transmuting the baser metals into gold. At the present time we of the medical profession claim to be the searchers, and indulge this appetite.

In our search for new remedies we are obliged to look backward and reflect upon the past, in order to know that we are really using a new remedy. We can only refer to a very few extensively used or positively useful remedies that will bear the test of *new*.

If we look up the history of one of the most extensively used remedies of the past ten years, viz: chlorate of potassa, we find it used for phagedena and cancrum oris in 1843, and its probable use extending much farther back than that time, and mostly for the same class of diseases that it is used for at the present time; yet many practitioners look upon this as a comparatively new remedy. It probably is one of our best remedies, and one which has very marked good effects in the class of diseases to which it is adapted. We can frequently obtain new insight to the progress of medical science by examining what has past, and instituting a comparison with the present. For instance, in examining the past we are led to ask what has become of that most constantly used, and on the part of the patient most commonly expected remedy, *blood-letting*, which was so fashionable thirty or forty years ago. It led the van in the list of remedies in all the older works on practice. Mackintosh, in his practice, says of this remedy, used in painter's colic, "free bleeding from the arm, in the first stages of the malady, can hardly be dispensed with;" while Watson, at the present time treating the same disease, says not one word of this "hardly to be dispensed with" remedy. Again, Dr. Mackintosh says of Dr. Alison's arguments against blood-letting in the cold stage of intermittent fevers that "such objections as he offers to the use of this remedy are whimsical, weak and fallacious;" while Watson very modestly says: "I have never seen *one* case where I thought such an heroic remedy was necessary in the cold stage." He furthermore says, "this remedy is not requisite *at all*, unless there is danger of some internal inflammation."

One of the next most active remedies of the older authors was that associate of blood-letting, *tartar emetic*, now ranked as one of moderate usefulness, and very unfrequently employed, even in many of the most inflammatory diseases. These active remedies have been superseded in a measure at the present day by an old remedy put to a new purpose, viz: *veratrum viridi*. This remedy as an arterial sedative since its first introduction into general use, about eight years since, has not been over-rated, and it is said to possess some alterative powers. But the indiscriminate use of a positively useful remedy will sometimes place it in peril of losing its usefulness. Thus it has been with this eminently beneficial medicine. While it has the power of controlling the action of the great centres of the circulatory apparatus, its depressing effect upon the nervous system under an improper dose is sometimes disastrous. The use of this remedy in active inflammatory diseases has become very general; still many of our profession report a want of manifest success in the effects desired to be obtained. I would say that in many instances I have felt that want of confidence in its use, while again I have seen the most marked benefit result from its exhibition.

The popular antipathy against the use of *calomel* has led to the abandonment of the very *liberal* and *persevering* employment of the different mercurial preparations, and a substitute of other remedies for them. For instance, in journalistic essays upon the treatment of syphilitic diseases, we find writers who enlarge upon the anti-mercurial treatment of syphilis ; while others, equally positive, contend for the treatment of all syphilitic disease with mercury in some form. So we have recommended as an anti-syphilitic remedy *iodide of potassium*, and various vegetable compositions, such as sarsaparilla, guiac wood, poke root, yellow dock, &c., mith their powers praised for eradicating this disease from the system.

Remedies are new when they are fashionable. This saying would hardly seem true, but we have only to examine the rise and fall of the drug market to find that it is so.

*Cod liver oil* a few years since served to absorb all the attention of doctors in the treatment of phthisis ; while cod liver oil at the present time is in a great measure unfashionable, and *hypophosphites of soda*, *lime* and *iron* are performing many wonderful changes in the arrest and improvement of this disease. But a few years since this disease was thought to have received a severe check, if not a curative specific, in chlorate of potassa ; while very recently almost all the written testimony in relation to the use of this remedy in phthisis agrees in its inefficiency. So that, in opposition to doctors and the "clergyman whose sands of life were so nearly run out," phthisis continues to be the door of exit to hundreds of thousands annually.

As one of the more recently used remedies, and one which would be considered nearly new, I would here mention *bromine* and its different compounds. Bromine has been quite largely employed during the recent contest between the North and South, in hospital practice. As an antiseptic and disinfectant it was used in the erysipelas wards of different hospitals. After this it was used as a remedy in chronic diarrhœa, a disease which proved quite obstinate and persistent under all varieties of treatment ; while under the use of bromine it is said to have been wonderfully successful. Bromine in the form of bromide of potassium has been in the list of remedies for several years. In 1858, one of the medical journals of the day notices an article from a French journal, written by M. Alf. Binet, recommending the use of this preparation of bromine in the treatment of spermatorrhœa, as employed by Dr. Thirlmann, a Russian physician. He says : "The sedative effect of this substance on the genital organs is well known, causing loss of virile power for several days after the medicine has been discontinued. M. Binet reports three obervations of spermatorrhœa in which the effect of the bromide was evident and rapid. The first patient had suffered for seventeen years from spermatorrhœa. He had several emissions every night. After the first dose the emissions were reduced to one nightly; at the end of a week they ceased to reappear only once, and in a month the patient left the hospital well. The subject of the second observation had been affected for several years. At the time the treatment was began he had two, three, and even five pollutions a night. An immediate improvement followed the administration of the medicine. After a fortnight the patient had only one emission every fourth day. In the third case the patient had several pollutions every night. After using the remedy six weeks he was cured, and discontinued the medicine. In a month he had a relapse; but treatment was resumed with the same success. The dose was from 10 to 30 grains. No ill effects were observed in either csae." I quote this extract as introductory to my *use* of the remedy for the same purpose. I commenced using it in practice soon after reading this notice of its employment by M. Binet, and have probably treated about a dozen cases with uniform success.

I was called upon about eighteen months since by Mr. S., suffering from involuntary seminal discharges—a young man of some 25 years of age. He stated his deplorable condition, his health being fast undermined, and mentally almost as imbecile as an idiot, which condition he certainly seemed fast approaching. Tears would flow at the talk of his condition. Under the promise of a persevering use of the remedies I might prescribe, he commenced the use of bromide of potassium in solution in the quantity of about ten grains daily. From the commencement of the use of this remedy his discharges diminished in frequency, his spirits revived, and hope began to encourage him in the belief that he might recover. His improvement was rapid, and in about three months he was able to labor, and in one year he was as stout and healthy a man as the farming community in which he lived could produce.

I might refer to other special cases; but my object is not so much to journalize cases as to give my general experience and observations in relation to the use of this medicine, which many may esteem new, and particularly so in its application to the above named disease. This remedy has likewise been employed as an anti-spasmodic in epilepsy, chorea, and other spasmodic affections, and by many with a very marked benefit.

I would recommend it to the profession as worthy a trial in the class of diseases referred to above.

In Braithwaite's Retrospect, Nos. 45, 46 and 47, for the years 1862 and 1863, we find several articles written concerning the use of *Sarracenia Purpurea* in variola, or small-pox. It comes to us in its medical history from an old Indian squaw residing in Nova Scotia, who had an extensive reputation for curing the small-pox.

Dr. Miles having from this source obtained a knowledge of its use, employed the remedy in an epidemic of small-pox at that time prevailing in Nova Scotia. He likewise received reports of its trial by other physicians to whom its use had been disclosed. One of these latter, a Dr. Richardson, of Toronto, Canada, writes: "On the whole I must conclude that the effect produced by the Sarracenia was most satisfactory and well marked. The disease continued very severe until it was administered, and became entirely changed in its severity after the administration of the third dose, the effect being due to it alone, as no other remedy was given after it was commenced. I am forced to the conclusion that the secondary fever was much controlled by it, and that dessication took place much more rapidly than would have occurred otherwise. Dr. Morris, of Halifax, Nova Scotia, contends, in an article in No. 47 of the Retrospect, for the priority of its introduction into use as a remedy in variola. He gives a full history of obtaining the secret of its use, and a botanical history of the plant, in connection with the method of its use and its effects.

I would represent to this Society that during the prevalence of small-pox in our city some two years since I made a trial of the Sarracenia, and was quite favorably impressed with its effects.

My first use of the article was a decoction of the dried root as recommended by Drs. Morris and Miles, and in those cases I thought the remedy had the effect to quiet the febrile excitement, and by the third or fourth day after the appearance of the eruption was impressed with the belief that the pustules were aborted, as they soon dried up, without the appearance of maturity. I think that most of the cases (some twenty in number) were much modified by the use of the *root*. Afterward, being unable to obtain the root alone, I used a decoction of the leaves, with but a small proportion of the root combined. With this decoction I could perceive no marked benefit. I think the remedy deserves a further trial and more close observation. For the trial, take the root most carefully prepared. For a remedy, with sure and beneficial effects, which will even modify, not to say prevent, a disease so much dreaded as that of small-pox, would be hailed with great joy by individuals, nations, worlds.

